# Perspectives and future directions on the prevention and control of emerging and re-emerging vector-borne and zoonotic diseases: findings from across the WHO Eastern Mediterranean Region

**DOI:** 10.1093/eurpub/ckae115

**Published:** 2025-01-13

**Authors:** Ziad Memish, Shahul H Ebrahim, Richard Brennan, Chiori Kodama, Sundus Haji-Jama, Thomas Mollet, Abdinasir Abubakar

**Affiliations:** College of Medicine, Alfaisal University, Medical & Humanitarian Research, King Salman Humanitarian Aid & Relief Center, Riyadh, Saudi Arabia; School of Public Health, University of Sciences, Technique, and Technology, Bamako, Mali; World Health Organization, Eastern Mediterranean Regional Office, Health Emergencies Programme, Cairo, Egypt; World Health Organization, Eastern Mediterranean Regional Office, Health Emergencies Programme, Cairo, Egypt; World Health Organization, Eastern Mediterranean Regional Office, Health Emergencies Programme, Cairo, Egypt; World Health Organization, Eastern Mediterranean Regional Office, Health Emergencies Programme, Cairo, Egypt; World Health Organization, Country Office for Lebanon, Beirut, Lebanon

Vector-borne and zoonotic diseases (VBZDs) represent a major global public health concern. Vector-borne diseases, caused by parasites, viruses, and bacteria transmitted by vectors, make up over 17% of infectious diseases, causing 700 000 deaths annually [1]. Zoonotic diseases, which spread between animals and humans, have become more prevalent, accounting for 60% of human infectious diseases and 75% of emerging infections [1]. They result in 2.5 billion cases and 2.7 million deaths worldwide each year [1].

The WHO Eastern Mediterranean Region (EMR) includes 22 countries: Afghanistan, Bahrain, Djibouti, Egypt, Islamic Republic of Iran, Iraq, Jordan, Kuwait, Lebanon, Libya, Morocco, Occupied Palestinian Territory, Oman, Pakistan, Qatar, Saudi Arabia, Somalia, Sudan, Syrian Arab Republic, Tunisia, United Arab Emirates, and Yemen. The region has faced significant threats from zoonotic, emerging, and vector-borne pathogens, such as COVID-19 and the 2009 H1N1 pandemics, but also Middle East respiratory syndrome coronavirus (MERS-CoV), Crimean- Congo haemorrhagic fever (CCHF), Dengue fever, Rift Valley fever (RVF), among many others. Around 40% of the global population in need of humanitarian aid resides in Eastern Mediterranean Region, with fragile health systems and inadequate disease surveillance, preparedness, and response capacities [1]. Factors such as international travel and trade, rapid urbanization, mass gatherings of people [2], mass movements of live animals [3], climate change, conflict and state fragility, and natural disasters, and close proximity of animal rearing to human settlements exacerbate the EMR's vulnerability, fostering an environment conducive to zoonotic illnesses and frequent outbreaks of emerging infectious diseases.

Despite ongoing efforts by WHO, Member States and partners to prevent and control such diseases, the burden of VBZDs in WHO’s EMR is on the rise, with significant disparities in data availability across countries. A recent review [1] on VBZDs in the EMR also concluded that available published and non-published information is fairly limited, particularly in complex humanitarian settings.

In 2023, WHO, the Food and Agriculture Organization (FAO), and the World Organisation for Animal Health (WOAH) convened a consultative meeting to develop strategic guidance for the prevention and control of emerging and re-emerging VBZDs in the WHO’s EMR [3]. A key recommendation from the meeting was the urgent need for more local and regional research to better understand the transmission dynamics and risk factors of VBZDs affecting both humans and animals, and to identify key research priorities for VBZDs in the region. In an effort to address these gaps, WHO EMRO issued a call for papers to encourage research and understanding on the burden of VBZDs, epidemiological trends, challenges, advancements, and innovative strategies in prevention, preparedness, detection, and response to VBZDs across the region.

This journal supplement is a collection of 10 original manuscripts, each offering valuable and unique contributions to our understanding of VBZDs in the EMR, including disease and vector epidemiology studies, diagnostics, outbreak response evaluations, and One Health collaborations. The supplement includes work that enlightens on the epidemiology, transmission dynamics, and risk factors of diseases, such as the ongoing Crimean-Congo hemorrhagic fever outbreak in Iraq [4] and rabies dynamics in the Arabian Peninsula [5]. Descriptive works cover the epidemiology and strategic responses to Dengue Fever, with example from Afghanistan [6], as well as Chikungunya in the region [7].

Disease importation dynamics are examined through a risk assessment of imported malaria in Qatar [8]. Two research studies report on agent-specific epidemiology: zoonotic bacterial agents in rodents and small mammals of Iran [9] and the prevalence of Bartonellosis in the region [10]. Basic science efforts are detailed in: molecular tools for diagnosing MERS-CoV in Jordan [11] and research on Aedes albopictus to identify suitable candidates for paratransgenesis [12]. Examples of national responses include multi-sectoral approaches to managing Rift Valley fever in Sudan [13].

The evidence presented in this supplement confirms the pressing need for a multi-sectoral, comprehensive and coordinated approach to address VBZDs in the EMR. Enhanced collaboration among policymakers, health professionals, and researchers as outlined in the One Health framework [14, 15] and adequate financial commitments can help advance disease surveillance, improve diagnostic capabilities, and implement effective prevention and control strategies.

The ongoing global VBZDs epidemics and outbreaks continue to pose a significant risk to EMR countries. The disproportionate burden and enablers in EMR [15], including mass gathering events [2], serves as an alert and an opportunity to leverage the knowledge and tools before reaching accelerated levels of spread and virological adaptations. By prioritizing research, fostering international cooperation, strengthening health systems, addressing climate change and health, we can make progress in preventing the onset of new outbreaks, reducing burden of existing diseases and improving the health and well-being of vulnerable populations.
